# Impact of the first Gulf war on multiple sclerosis risk in Kuwait: a quasi-experimental study

**DOI:** 10.1186/s12883-023-03295-3

**Published:** 2023-07-05

**Authors:** Saeed Akhtar, Jasem Y. Al-Hashel, Raed Alroughani

**Affiliations:** 1 Department of Community Medicine and Behavioural Sciences, College of Medicine, Kuwait University, PO Box 24923, Safat, 13110 Kuwait; 2 Department of Medicine, College of Medicine, Kuwait University, PO Box 24923, Safat, 13110 Kuwait; 3 Department of Neurology, Ibn Sina Hospital, Kuwait City, Kuwait; 4 Division of Neurology, Department of Medicine, Amiri Hospital, Arabian Gulf Street, Sharq, 13041 Kuwait

**Keywords:** Interrupted time series design, Multiple sclerosis, Risk, First Gulf War, Impact assessment, Kuwait

## Abstract

**Objective:**

It has been reasoned that stressful life events tend to alter immune function thereby increasing the susceptibility to autoimmune diseases including multiple sclerosis (MS). Using the database of Kuwait National MS Registry, this quasi-experimental study assessed the impact of the first Gulf War (Iraqi invasion of Kuwait in 1990) on MS risk in Kuwait.

**Methods:**

MS incidence data from 1980 to 2019 were obtained from the Kuwait National MS Registry. Annual age-standardized incidence rates (ASIRs) (per 10^5^ person-years) were computed using the World Standard Population as a reference. Interrupted time series analysis with the option of autoregressive order (1) was used to evaluate the impact of the first Gulf War on MS risk by treating 1990 as an intervention year.

**Results:**

Estimated baseline annual ASIR (per 10^5^ person-years) was 0.38 (95% CI: -1.02, 1.78; *p* = 0.587). MS ASIRs (per 10^5^ person-years) tended to increase significantly every year prior to 1990 by 0.45 (ASIR per 10^5^ person-years = 0.45; 95% CI: 0.15, 0.76; *p* = 0.005). During the first year of the first Gulf War, there seemed to be a non-significant increase (step change) in ASIRs (per 10^5^ person-years) of MS (ASIR per 10^5^ person-years = 0.85; 95% CI: − 5.16, 6.86; *p* = 0.775) followed by a non-significant increase in the annual trend in MS ASIRs per 10^5^ person-years (relative to the preintervention trend i.e., the difference between the pre-first Gulf War versus the post-first Gulf War trends) by 0.65 (ASIR per 10^5^ person-years = 0.65; 95% CI: − 0.22, 1.52; *p* = 0.138). However, a postestimation measure of the post-first Gulf War trend was statistically significant (ASIR per 10^5^ person-years = 1.10; 95% CI: 0.40, 1.80; *p* = 0.003), which implies that the post-first Gulf War trend in the annual ASIRs (per 10^5^ person-years) inclined to be the same as was the pre-first Gulf War (i.e., counterfactual of the pre-first Gulf War trend in annual ASIRs (per 10^5^ person-years) as if no first Gulf War took place).The Durbin-Watson test statistic (*d* = 1.89) showed almost non-significant autocorrelations across the time series observations on ASIRs (per 10^5^ person-years).

**Conclusions:**

This study suggests that the first Gulf War was not significantly associated with the increasing trend in MS risk at population level in Kuwait neither with any short-term change nor with secular trend. Future studies may consider confirming the role of conflict-related stress or other stressful life events in potential exacerbation of MS risk along with unraveling biologically plausible mechanistic pathways.

## Introduction

Multiple sclerosis (MS) is one of the world’s most common neurologic disorders characterized by inflammation and degeneration of both myelin and axons in central nervous system [1]. The estimated number of people with MS increased from 2.1 million in 2008 to 2.3 million in 2013 worldwide [2]. Among young adults in many countries, it is the leading cause of non-traumatic neurologic severe disability and related socio-economic burden [1, 3, 4]. Globally, the MS prevalence ranges between 2 and 150 per 100,000 with an increasing trend [5]. Despite decades of intensive research, no specific cause for this disorder has been identified. Epidemiologic studies have suggested the role of genetic and non-genetic risk factors in MS etiology including familial clustering, exposure to infections, chemical and physical agents, vaccinations, hormonal factors, dietary patterns, occupation, environment, physical trauma and psychological stress [6–8]. Disparities in environmental risk factors and genetic predispositions modulate the MS risk at population level, thus contributing to a wide global variation in MS risk. Furthermore, the stressful life events may alter immune function and affect susceptibility to autoimmune diseases including MS [9]. Several studies have demonstrated that stressful life events might have an adverse effect on the MS risk [10–13]. Though, the mechanisms by which such stressors trigger pathogenetic events in MS hitherto are unknown [11, 13, 14]. Nonetheless, the published epidemiological data examining the role of stress in MS remain inconsistent [13, 15–17].

Based on the Kurtzke classification Arabian Gulf Region of the Middle East including Kuwait was considered as a low-risk zone for MS. However, subsequent studies suggest a moderate-to-high prevalence (31–55 MS cases per 100,000 population), with an increasing MS incidence trend [18]. Kuwait also experienced a marked rise in the MS prevalence (per 100,000 population) from 2011 (85.1) to 2018 (104.9), and this increase was more evident in females (108.9 to 137.1) than males (60.8 to 71.2). Additionally, MS annual incidence (per 100,000 population) in Kuwait showed an upward trend, from 2003 (2.3) to 2017 (5.4) and the corresponding estimates were more pronounced in females (2.8 to 7.0) than males (1.8 to 3.8) [19, 20]. This steady increase in prevalence and incidence has been attributed to one or more of the following; i.e., a true increase in MS prevalence worldwide, an increase in public awareness, change in socioeconomic status, improvements in health-care systems, introduction of the national MS registry, improvement in the accessibility to medical facilities and the availability of magnetic resonance imaging (MRI) in parallel with the introduction of revised versions of diagnostic criteria [19].

Historically, post-war MS epidemics have been reported in the Faroe Islands and Iceland occupied by foreign troops [21, 22]. The United States veterans of World War II, the Korean War, Vietnam and the first Gulf War (Iraqi invasion of Kuwait in 1990) experienced a distinct, elevated increase in MS risk compared with the general US population [23–25]. These findings suggest that both a racial and a possibly genetic predisposition, as well as a geographically determined differential exposure to an environmental agent related to MS risk [1, 23, 26, 27]. A population-based case-control study has also identified that being resident/presence in Kuwait at the time of Iraqi invasion during the first Gulf War was a significant risk factor for increased odds of being a MS case [28]. However, Al-fasy and colleagues did not account for the duration of stay in the country during Iraqi invasion and occupation, since many nationals and expatriate workers left the country during that time. Furthermore, the previous studies in Kuwait have shown a steadily increasing tendency in age-standardized rates of MS by calendar year and sex [19, 20, 29] with disproportionate increase in females than males with sex ratio (female: male) of 1.9 (range 0.4-3.0) [30]. In this study, we sought to examine whether the first Gulf War was associated with an increased MS risk in Kuwait more than what was expected after accounting for the secular component of series of the annual age-standardized incidence rates (ASIRs) (per 100,000 person-years) by using an interrupted time-series (ITS) design.

## Methods

### Study design, setting, and population

We used the ITS design to assess the immediate and long-term impact of the first Gulf War on the MS risk in Kuwait. Kuwait is small oil-producing country and is located between latitudes 28° 45′ and 30° 05′ N and longitudes 46° 30′ and 48° 30′ E at the northwest corner of the Arabian Gulf. The state is bounded by Iraq to the north and west and Saudi Arabia to the south. Migrant workers constitute about 70% of the population, and resultantly, 80% of the labor force comprises migrants [31]. These migrants originate predominantly from Southeast Asia, Eastern Mediterranean and African regions with varying range of MS incidence and prevalence [3, 32]. There is large turnover of these workers; every year thousands of them leave and new ones arrive in Kuwait. Of the migrants, 46% are 20 to 44-year-old and predominantly live as single, mainly because of their inability to fulfil a legal requirement of minimum wages to be able to bring their families [33]. Health services are free for all the citizens and the residents are obligated to pay nominal cost for the required health services in Kuwait. Among Kuwaiti nationals, the male and female literacy rates (defined as the individuals aged 10 years or more who can read and write) are 98.5% and 91.2%, respectively, with a sex ratio (male:female) of 1:1.04 at birth [34]. Administratively, Kuwait has six governorates. Medical services in each governorate comprise a network of primary healthcare clinics and a public hospital. Additionally, there are centralized specialty hospitals, which serve patients from the entire country. Moreover, Kuwait has national-level specific disease registries such as Kuwait Cancer Control Center and Kuwait National MS Registry (KNMSR).

### Data sources

We used the MS patients’ data from the KNMSR. The variables included were current age (years), age (years) at MS diagnosis, sex (female or male), nativity (Kuwaiti or non-Kuwaiti). The MS confirmatory diagnosis and registration of the incident MS cases in KNMSR have been described in detail elsewhere [31, 35, 36], and briefly outlined here. KNMSR was established in 2010 after combining the databases of all the major hospitals including neurology clinics along with the main MS clinics at Dasman Research Institute and Ibn Sina Hospital, which together accounted for nearly 98% of the MS patients diagnosed in Kuwait. Initial and follow-up assessment sheets were provided to all the hospitals and neurology clinics and were completed by the neurologists. The acquired data were cross-checked and entered by neurologists experienced in diagnosis and treatment of MS patients. Prior to the application of the revised 2010 McDonald criteria for MS diagnosis used in the registry [37], confirmatory MS diagnoses by the treating neurologists were based on the earlier accepted diagnostic criteria [38, 39]. Once included in the registry, patients were followed prospectively on a regular basis (at least once every 6 months), and their clinical data were updated in the registry. Since its establishment, the KNMSR database is being managed and updated on a monthly basis. Kuwait’s population data by age, sex, nativity, and calendar year were obtained from the Public Authority for Civil Information, Ministry of Interior, Kuwait.

### Statistical analysis

We computed the annual ASIRs (10^5^ person-years) of MS using the World Standard Population as a reference [40]. ITS analysis was conducted to evaluate an immediate level (step change) and long-term impact of the first Gulf War on the MS risk in Kuwait using meticulously collected and managed National MS Registry data from January 1980 to December 2019. This analysis can validate whether the first Gulf War influenced MS risk that was significantly greater than the underlying trend in the data collected repetitively overtime before and after the first Gulf War (a discrete event). Another notable strength of ITS analysis of observational data in examining the impact of an identifiable discrete event (intervention) is that it enables to control for the effect of pre-intervention secular trends in a time series of outcome measures [41, 42]. We therefore undertook ITS analysis of a series of population level annual ASIRs (per 10^5^ person-years) over a period from 1980 to 2019 and interrupted by the first Gulf War. We used this period because of the availability of meticulously collected and managed National MS Registry data for this period. We fitted a generalized linear regression model of the form: $E{(Y}_{t})={\beta }_{0}+ {\beta }_{1}{T}_{t}+ {\beta }_{2}{X}_{t}+{\beta }_{3}{{X}_{t}T}_{t}+{\epsilon }_{t}$*where*: ${Y}_{t}$ = annual ASIR (per 10^5^ person-years) of MS; ${T}_{t}=$ time since the beginning of the study until time *t*; ${X}_{t}$= an indicator variable representing the first Gulf War (coded as 0 for pre-first Gulf War years and 1 for the year 1990 and subsequent years); ${{X}_{t}T}_{t}$ = interaction between time and indicator variable for first Gulf War; ${\epsilon }_{t}$ = residual at time *t;*${\beta }_{0}=$ baseline annual ASIR (per 10^5^ person-years) of MS; ${\beta }_{1}=$ pre-first Gulf War slope; ${\beta }_{2}=$ step (level) change in the annual ASIR (per 10^5^ person-years) trend immediately post-first Gulf War compared with the expected counterfactual (continuation of pre-first Gulf War slope as if no first Gulf War took place); ${\beta }_{3}$ = difference between pre-first Gulf War versus post-first Gulf War trends. Furthermore, using the model coefficients, a postestimation measure was computed as the linear combination (${\beta }_{1}+{\beta }_{3})$ of pre-first Gulf War trend (${\beta }_{1}$) and the difference between pre-first Gulf War and post-first Gulf War trends (${\beta }_{3})$ to reflect the post-first Gulf War trend.

Briefly, this model accounted for the underlying trend in the annual ASIRs (per 10^5^ person-years) of MS and provided the estimates of pre-first Gulf War trend, immediate (step level) change after first Gulf War and post-first Gulf War gradual change (slope) over time relative to pre-first Gulf War trend (slope). Immediately following the first Gulf War, the level (step) change in the annual ASIRs (per 10^5^ person-years) of MS was modelled using an indicator variable (${X}_{t}$) for first Gulf War and the slope change was modelled using an interaction (${{X}_{t}T}_{t}$) between the indicator variable for first Gulf War and the time (years as a continuous variable) to reflect a post-first Gulf War trend in annual ASIRs (per 10^5^ person-years). To account for the autocorrelations, a first-order autoregressive (AR1) model was fitted. We used the residual analysis to evaluate the adequacy of the resulting model. First-order autocorrelation was statistically assessed by using Durbin-Watson (DW) test statistic (*d*). The computed DW statistic *d* is used to evaluate autocorrelation in the residuals from a regression model. DW statistic *d* can take on values between 0 and 4. Under the null hypothesis (H_o_: autocorrelations = 0), DW statistic *d* takes on the value of 2. The values of *d* less than 2 suggest positive autocorrelation, whereas values of *d* greater than 2 suggest negative autocorrelation between a residual at time *t* and the residuals prior and/ or subsequent to it in the time-series. The higher-order autocorrelations were evaluated using autocorrelation and partial autocorrelation functions. We computed 95% confidence intervals (CI) using semi-robust standard errors. Statistical significance of regression coefficients was determined with two-tailed *t*-tests [41]. All the analyses were conducted using Stata 17.0/ SE (Stata Corp LLC, Texas, USA). The patients with MS initially gave their informed consent for inclusion in the KNMSR and the use of their deidentified information for research purposes. The study was approved by the Research Committee of the Audit and Planning Office, Ministry of Health, Kuwait under a decree of 1670/2021. Furthermore, the study was also approved by the Ethics Committee, Health Sciences Center, Kuwait University. This study was undertaken in accordance with the principles and guidelines of the Declaration of Helsinki for medical research involving human subjects. Furthermore, the methods including design, conduct, data collection, analysis and reporting of the results conformed with the STROBE (Strengthening the Reporting of Observational Studies in Epidemiology) guidelines.

## Results

### Participants’ characteristics and basic statistics

**Fig. 1:**
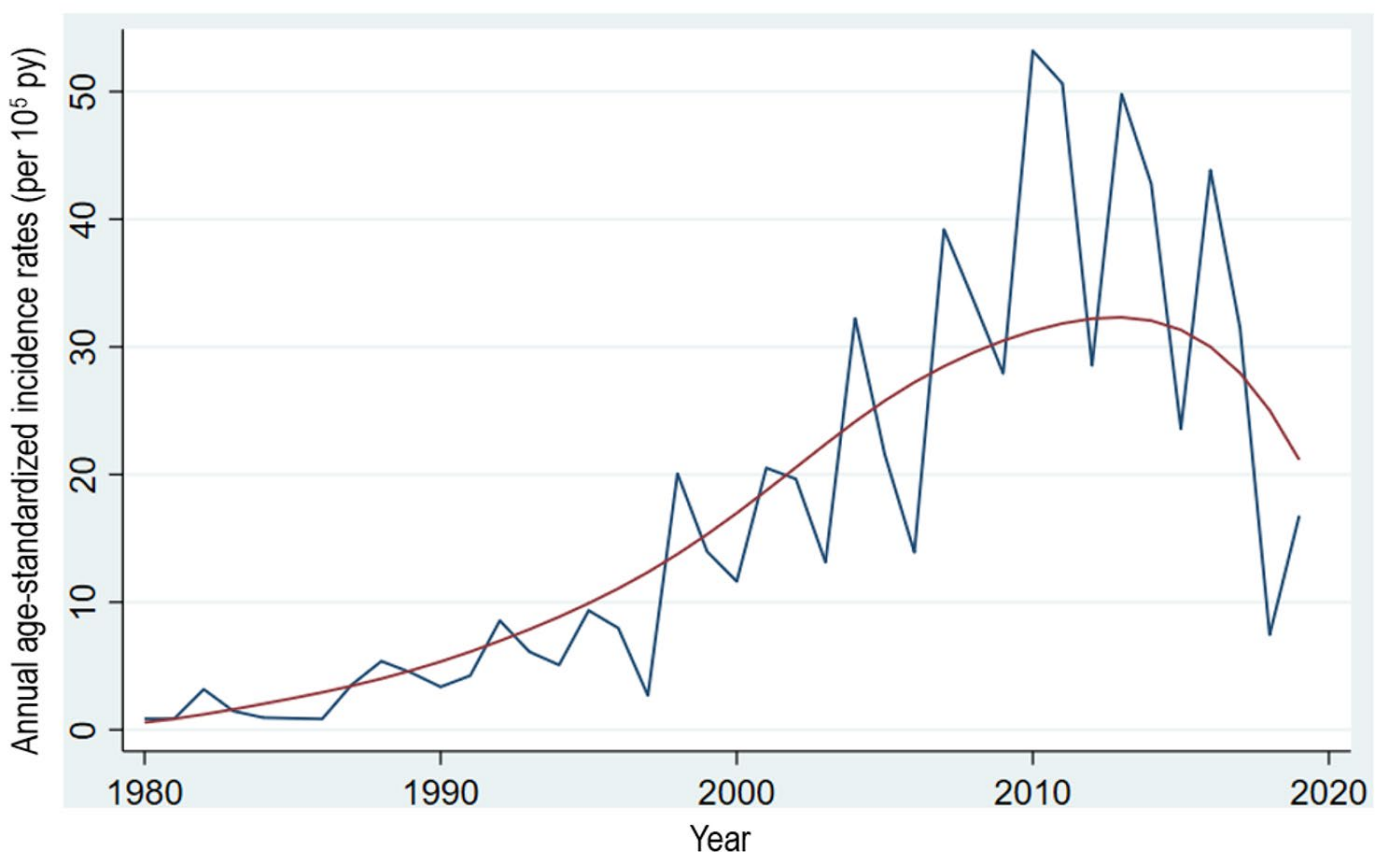
Line and loess (locally estimated scatterplot smoother) plots of annual age-standardized incidence rates (per 105 person-years) of multiple sclerosis in Kuwait:1980-2019

During the study period from January 1980 to December 2019, 1764, MS cases of 95.6 million person-years at-risk were diagnosed. Of the MS patients, 1,506 (85.4%) had relapsing-remitting MS course, followed by 138 (7.8%) patients with secondary progressive MS course, 63 (3.6%) with primary progressive MS course, and 57 (3.2%) patients were regarded as having clinically isolated syndrome. In the subsequent analysis, all the MS cases with the four subtypes were pooled as MS patients. Of the MS patients, 87% were Kuwaiti, 66.9% were females (sex ratio: 2.02), 71.5% were 20–39 years old, followed by 18.2% in age range of 0–19 years and 10.3% were 40 years old or older. The overall mean annual ASIR (10^5^ person-years) was 3.41 (95% CI: 1.61, 5.21) during the study period. The annual ASIRs (per 10^5^ person-years) are displayed as the line and loess (locally estimated scatterplot smoother) plots in Fig. 1. The mean annual ASIRs (per 10^5^ person-years) (95% CI) for eight 5-year periods showed an overall increasing trend till 2010-14 period followed by a slight dip in the last 5-year study period (Table 1).

**Table 1:** Mean annual age- and sex-standardized incidence rates (per 105 person-years) of multiple sclerosis for eight 5-year periods in Kuwait:1980–2019

Study period	Mean annual age- and sex-standardized incidence rates (per 10^5^ person-years)	95% confidence interval	
		Lower limit	Upper limit
1980-84	4.4	2.0	6.8
1985-89	6.2	0.6	11.8
1990-94	16.3	0.5	32.6
1995-99	23.8	1.4	46.2
2000-04	38.3	5.9	70.6
2005-09	59.3	14.5	104.0
2010-14	76.4	11.0	141.9
2015-19	48.3	7.9	88.6

## Interrupted time-series model

**Fig. 2:**
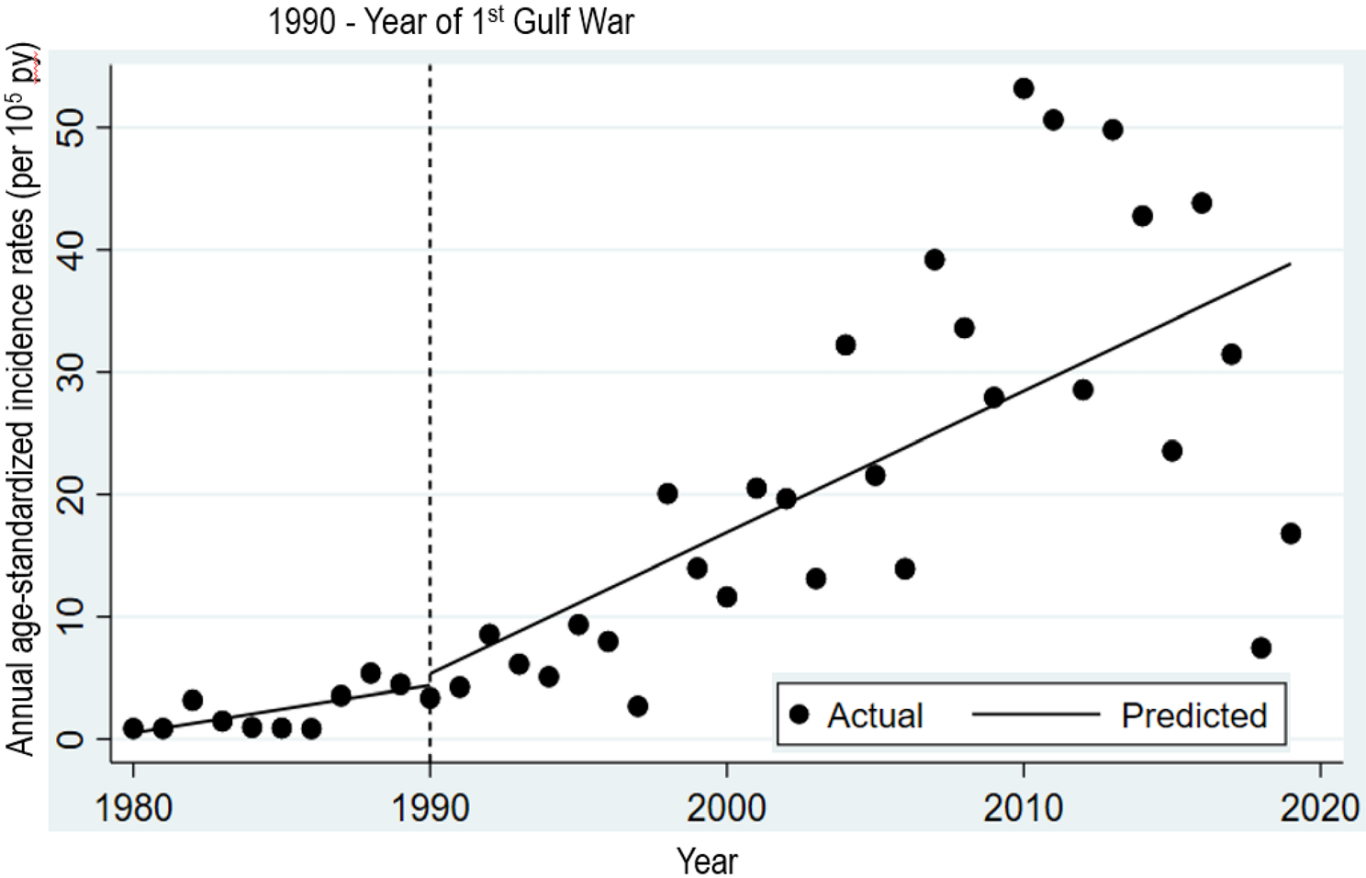
Interrupted time series analysis of annual age-standardized incidence rates (per 105 person-years) of multiple sclerosis in Kuwait:1980-2019

ITS analysis showed that the estimated baseline annual ASIR (per 10^5^ person-years) was 0.38 (${\beta }_{0}=0.38;$95% CI: -1.02, 1.78; *p* = 0.587). Annual ASIRs (per 10^5^ person-years) of MS tended to increase significantly prior to first Gulf War by 0.45 (${\beta }_{1}$= 0.45; 95% CI: 0.15, 0.76; *p* = 0.005). In the first year of post-first Gulf War, there seemed to be a non-significant immediate increase (level change) in the annual ASIRs (per 10^5^ person-years) of MS (${\beta }_{2}$ = 0.85; 95% CI: -5.16, 6.86; *p* = 0.775) followed by a non-significant increase in the post-first Gulf War trend relative to the pre-first Gulf War trend in the annual ASIRs (per 10^5^ person-years) of MS (${\beta }_{3}$= 0.65; 95% CI: − 0.22, 1.52; *p* = 0.138). However, a postestimation measure of post-first Gulf War trend was statistically significant (ASIR per 10^5^ person-years = 1.10; 95% CI: 0.40, 1.80; *p* = 0.003) (Table 2; Fig. 2). Which implies that the post-first Gulf War trend in annual ASIRs (per 10^5^ person-years) inclined to be the same as was the pre-first Gulf War (i.e., counterfactual of pre-first Gulf War trend in annual ASIRs (per 10^5^ person-years) as if no first Gulf War took place). The DW test statistic (*d* = 1.89) indicated that the first-order autocorrelations across the time series of ASIRs (per 10^5^ person-years) were negligible in magnitude.

**Table 2:** Parameters’ estimates of trend components identified in interrupted time-series analysis of the impact assessment of the first Gulf War of 1990 (first Gulf War) on MS risk in Kuwait:1980–2019

Description of parameter	Parameter’ estimate	95% CI***	*p*-value
Initial level of ASIRs (per 10^5^ py**) (*β*_*0*_)	0.380	− 1.024–1.784	0.587
Pre-first Gulf War linear trend in annual ASIRs (per 10^5^ py) (*β*_*1*_)	0.452	0.147–0.756	0.005
Immediate effect (step change) in post-first Gulf War linear trend in annual ASIRs (per 10^5^ py) (*β*_*2*_)	0.853	− 5.156–6.862	0.775
Difference in pre-first Gulf War trend vs. post-first Gulf War linear trend in annual ASIRs (per 10^5^ py) (*β*_*3*_)	0.652	− 0.221–1.525	0.138
Post-first Gulf War linear trend in ASIRs (per 10^5^ py) (*β*_*1*_ *+ β*_*3*_)	1.104	0.404–1.804	0.003

* ASIR = Age-standardized rate (per 10^5^); ** py = person-years; *** CI = Confidence interval

## Discussion

The risk factors’ pattern for MS is quite complex and multidimensional. Genetic and environmental factors have been implicated in MS causation, and such effects may modulate the age at MS onset and overall MS incidence. According to the infectious etiology of MS, infection with Epstein-Barr virus (EBV) has been conjectured to contribute to the MS risk. Mounting empirical evidence supports the pivotal role of the EBV in the development of MS [43]. Recently, it has been shown that compared with other viral infections, MS risk increased multifold after EBV infection [44, 45]. Environmental factors including vitamin D deficiency tend to influence the MS risk at two distinct age periods. However, the precise age at which the changes in vitamin D metabolism may increase the predisposition to MS particularly among women is still unknown [46]. Furthermore, latitude gradient has been linked with MS risk. Reportedly MS occurs more frequently in higher latitudes than in the places close to equator both in northern and southern hemispheres. The latitude gradient in MS incidence indicates low sun exposure, and the resultant vitamin D deficiency has been linked to increased MS risk [47, 48]. In contrast, some other studies reported no role of latitude gradient in MS causation [49, 50]. The Arabian Gulf Region is regarded as a low- to medium-risk zone for MS. However, the published data suggest a moderate- to-high MS prevalence (31–55 cases per 10^5^ population) in this region [18]. Kuwait is in the northern hemisphere with its midpoint approximately 3259 km (2025 miles) north of the equator, thus positioned in a low-to-medium MS risk zone. However, Kuwait with the known level of MS prevalence (85.1 MS patients per 10^5^ persons) is regarded as a high-risk country [19].

It has been debated that stressful life events may alter immune function and affect susceptibility to autoimmune diseases including MS. However, the published data from epidemiological investigations examining the role of stress in MS risk remain inconsistent [11, 13]. Furthermore, the mechanism(s) by which such an event(s) triggers pathogenetic mechanism for increasing MS risk is unclear [13, 15–17]. This study assessed the impact of the first Gulf War on MS risk in Kuwait using a quasi-experimental study design.

### Main findings

The estimated baseline level of annual ASIR (per 10^5^ person-years) of MS was 0.38. MS ASIRs tended to increase significantly every year prior to 1990. During the first year of the first Gulf War, there was a non-significant increase (level change) in annual ASIRs (per 10^5^ person-years) of MS. The post-first Gulf War trend relative to the pre-first Gulf War trend in annual ASIRs (per 10^5^ person-years) of MS (i.e., difference between pre-first Gulf War vs. post-first Gulf War trends) was also statistically non-significant. However, a linear combination of (i) pre-first Gulf War trend and (ii) the difference between pre-first Gulf War vs. post-first Gulf War trends as a postestimation measure showed that the post-first Gulf War trend in annual ASIRs (per 10^5^ person-years) sustained significantly as almost a continuation of pre-first Gulf War trend (counterfactual) without a significant first Gulf War effect on MS risk.

### Comparison with other studies

In this study, the immediate level (step) change and the change (difference) in the levels of slopes between post-first Gulf War relative to pre-first Gulf War was statistically non-significant, which showed that the short-term effect of the first Gulf War on MS risk was unimportant. We are unaware of any published data that had looked at the frequency of incident MS cases recorded soon after the initiation of a war and attributed to the war-related stress. Further investigations are needed to unravel the relationship between conflict-related stress and MS incidence along with untying mechanistic pathways.

The computed postestimation measure (𝛽_1_ + 𝛽_3_) as the post-first Gulf War trend in the annual ASIRs (per 10^5^ person-years) was statistically significant. This measure showed that despite the interruption in the time series during 1990 (i.e., population exposure to the first Gulf War), a significant upward trajectory in the annual ASIRs (per 10^5^ person-years) of MS continued in subsequent period without a meaningful first Gulf War effect and mirrored the counterfactual trend (i.e., continuation of pre-first Gulf War trend as if no first Gulf War took place). This finding of consistently increasing pre-first Gulf War trend without meaningful impact of first Gulf War on MS risk in Kuwait may be ascribed to steadily evolving improved diagnostic tools, cumulative number of specialized neurologists, progressively growing disease awareness and enhanced MS case-ascertainment. Previously, in this region, arguably the ‘Islamic Revolution’ (regarded as a stressful event at a population-level) has been linked to an increased MS risk in Iran and this MS risk was higher among women than men [51]. However, it was counterargued that this increased MS risk in Iran was not necessarily associated with stressful event of the Islamic Revolution’ rather mostly attributed to changed lifestyles among women in post-revolution era, wherein by law women were required to wear a veil in public, thereby minimizing exposure to Sun and the resultant vitamin D deficiency led to an increased MS risk. Nevertheless, it was debated that although this observation was important, it might not fully explain an increased MS risk in Iran. Since, in Iran during recent decades, better diagnostic tools, increased number of physicians, enhanced disease awareness and improved case-ascertainment were considered to have contributed in the observed increase in the MS risk [52].

By contrast however, a previous study in this setting showed that being in the country during the first Gulf War was significantly associated with increased odds of being a MS case than a control [28]. The finding of this study is also in contrast to the results of the previous studies based on the data from the United States veterans of World War II, Korean War [23, 53] and a postwar epidemic in Iceland [21], which, together showed somewhat elevated MS risk in this subpopulation relative to the general population of the United States [25, 54]. Furthermore, historically, it was claimed that the invasion and five-year occupation of Faroe Islands by British troops during the World War II introduced a widespread, specific, persistent (but unknown) and probably mild asymptomatic infection, as only one in 500 exposed individuals were clinically affected with primary MS [55]. However, subsequently only a small proportion of those affected with primary MS developed clinical signs. Afterward MS risk diminished across these Islands. However, authors’ claim as unknown transmissible infectious aetiologic agent of MS was speculative and unsubstantiated with laboratory diagnosis [22]. These varying results between non-significant influence of the first Gulf War on MS risk in Kuwait and slightly elevated MS risk elsewhere reported by earlier studies might be related to the difference in studied populations, diagnostic criteria for MS and statistical methods used. In the light of these conflicting reports, further studies are needed to explore the effect of war-related stress on MS risk and/ or exacerbation of MS along with the disentanglement of plausible mechanistic pathways for such relationships.

### Study strengths and limitations

This study has some strengths; (i) to the best of our knowledge, this is the first population-based study, which has empirically evaluated the impact of first Gulf War on MS risk using a national-level MS database in Kuwait; (ii) we analyzed a dataset which had a large number of time-series observations on annual ASIRs (per 10^5^ person-years) of MS before and after the first Gulf War which augmented the ITS analysis to have sufficient study power [56]; (iii) A feature of ITS analysis was that it took into account background secular trend in annual ASIRs (per 10^5^ person-years) of MS, while analyzing the first Gulf War effect on MS risk on long-term basis; (iv) Since, presumed first Gulf War effect on MS risk was unintended outcome, therefore, ITS analysis was a best suited technique in this study, wherein randomized control trial was infeasible to evaluate the relationship of first Gulf War and the stated outcome.

Following limitations of this study should be considered while interpreting the results; *First*, our inability to account for the potential influences of progressively increasing number of neurologists, improved diagnostic facilities over the decades. However, we contend that the influences of such health system improvements would have been uniform before and after the first Gulf War. *Second*, during the first Gulf War, there was a temporary disruption of health services in Kuwait due to exodus of migrant physicians and support staff to their native countries, which might have reduced the number of diagnosis and reporting frequency of MS cases in Kuwaiti nationals as well as migrant residents in Kuwait. *Third*, during the first Gulf War, many migrant workers returned to their home countries either temporarily or permanently, such a demographic shift could have influenced the MS diagnosis and case registration. However, we do not have empirical data to substantiate this contention. *Fourth*, various risk factors for MS including conflict-related stress have induction and latency periods, mostly of unknown lengths. Thus, if there were any stress-related influence on MS risk, due to unknown lengths of induction and latency periods, such an effect remained unverifiable in this study. *Fifth*, in our ITS analysis, we did not have a control population in the study, which would have strengthened the study conclusion compared to ITA analysis of the data on a single group which led to the absence of counterfactual outcome. *Sixth*, in this study, 18.5% MS patients were ≤ 19 years of age. This unusual frequency of MS patients in this age bracket might be a result of delay in presentation/ diagnosis. A delay of mean (SD) duration of 1.7 (2.9) years in MS diagnosis has been previously reported in Kuwait [57]. Public health authorities may consider accelerating their efforts for increasing awareness of neurological disorders among masses in the country. *Lastly*, the results of ITS analysis of annual ASIRs (per 10^5^ person-years) of MS do not lend themselves to make inference at the individual level, since the time series of MS ASIRs (per 10^5^ person-years) at a population level were analyzed.

## Conclusions

Taken together the results of this study suggest that the first Gulf War was not significantly associated with the increasing trend in MS risk at a population level in Kuwait neither with any short-term change nor with the secular trend. Future studies may consider confirming the role of conflict-related stress or other stressful life events in potential exacerbation of MS risk along with unraveling biologically plausible mechanistic pathways.

## Data Availability

The datasets generated and/or analyzed during the current study are not publicly available but are available from the corresponding author on reasonable request and with further approval of the Director, KNMS Registry.

## Declarations

### Authors’ Note

The abstract of this study was presented as a poster in Annual Conference of Society for Epidemiologic Research (USA) held at Chicago IL, USA on June 14–17, 2022.
